# Identifiers.org: Compact Identifier services in the cloud

**DOI:** 10.1093/bioinformatics/btaa864

**Published:** 2020-12-11

**Authors:** Manuel Bernal-Llinares, Javier Ferrer-Gómez, Nick Juty, Carole Goble, Sarala M Wimalaratne, Henning Hermjakob

**Affiliations:** European Bioinformatics Institute (EMBL-EBI), European Molecular Biology Laboratory, Wellcome Genome Campus, CB10 1SD Cambridge, UK; European Bioinformatics Institute (EMBL-EBI), European Molecular Biology Laboratory, Wellcome Genome Campus, CB10 1SD Cambridge, UK; Department of Computer Science, University of Manchester, M139PL Manchester, UK; Department of Computer Science, University of Manchester, M139PL Manchester, UK; European Bioinformatics Institute (EMBL-EBI), European Molecular Biology Laboratory, Wellcome Genome Campus, CB10 1SD Cambridge, UK; European Bioinformatics Institute (EMBL-EBI), European Molecular Biology Laboratory, Wellcome Genome Campus, CB10 1SD Cambridge, UK

## Abstract

**Motivation:**

Since its launch in 2010, Identifiers.org has become an important tool for the annotation and cross-referencing of Life Science data. In 2016, we established the Compact Identifier (CID) scheme (prefix: accession) to generate globally unique identifiers for data resources using their locally assigned accession identifiers. Since then, we have developed and improved services to support the growing need to create, reference and resolve CIDs, in systems ranging from human readable text to cloud-based e-infrastructures, by providing high availability and low-latency cloud-based services, backed by a high-quality, manually curated resource.

**Results:**

We describe a set of services that can be used to construct and resolve CIDs in Life Sciences and beyond. We have developed a new front end for accessing the Identifiers.org registry data and APIs to simplify integration of Identifiers.org CID services with third-party applications. We have also deployed the new Identifiers.org infrastructure in a commercial cloud environment, bringing our services closer to the data.

**Availabilityand implementation:**

https://identifiers.org.

## 1 Introduction

The Identifiers.org registry contains manually curated, high-quality metadata for hundreds of data collections, mostly in the Life Science domain (Juty *et al*., 2013), with each assigned a unique namespace, which can be used as a prefix (Wimalaratne *et al*., 2018). Other information stored includes a description of the data collection (the set of resources providing the data) and a formal definition of its identifier pattern [allowing Compact Identifier (CID) validation]. For each hosting resource, information is stored regarding institutional details, geo-location, hosted access URL, and whether it is the acknowledged primary provider of the data in question. Automated checks are performed registry wide on all the listed resources, to determine their availability. Identifiers.org provides Uniform Resource Identifier (URIs) based on the namespace assigned, for example: https://identifiers.org/taxonomy:9606. These namespaces are also used as prefixes for human readable CIDs (PREFIX: ACCESSION), for example: taxonomy:9606. An additional layer of prefixes is also assigned at the provider level (PROVIDER_CODE), enabling acknowledgement of, and resolution, at individual resources. For example, ncbi/taxonomy:9606 will resolve to the ‘taxonomy’ collection as hosted at the National Center for Biotechnology Information (NCBI). However, we recommend using CIDs without PROVIDER_CODE where possible. More recently, the use of CIDs has been recommended by Nature Scientific Data for data citations (Editorial, 2018), and by Google Dataset Search for identifiers (https://developers.google.com/search/docs/data-types/dataset). Historically, resolving systems addressing Life Science data records have focused on a simple one-to-one redirection of identifier to a resolving location (Smith *et al*., 2007), even where information on multiple resolving locations is known (Juty *et al*., 2013). The Identifiers.org resolution system addresses this limitation through acknowledging (in the data model) equivalent data availability in multiple locations (Juty *et al*., 2013), and therefore provides a consistent way to access and cite specific databases and individual providers. To facilitate international interoperability and provide backup redundancy, Identifiers.org collaborates with the US-based Name-to-Thing (N2T, https://n2t.net/) resolver to harmonize the resolution of CIDs (Wimalaratne *et al*., 2018). N2T is hosted by the California Digital Library, serving all ten of the University of California campuses.

The Identifiers.org system has been running on an EMBL-EBI production-level datacenter, with fallback facilities, over the past ten years. However, relying on one datacenter located centrally does not support the Identifiers.org resolver service use case optimally. This may incur a latency penalty, as well as introducing a dependency due to a single point of failure. Furthermore, an increasing number of Life Science resources are moving their data and tools into cloud environments to provide better availability and more robust scalability. Here, we describe the new Identifiers.org cloud-based architecture and services to generate and resolve CIDs, highlighting the benefits of a cloud-based system.

## 2 Materials and methods

The new cloud native Identifiers.org platform is based on a microservices architecture and is split in two different deployments: (i) a Central Registry and services; (ii) Distributed Resolution services.

The Central Registry, which contains all the information on namespaces, resources, their locations, etc. is centrally managed in a Post-greSQL data backend, and it provides the Distributed Resolution services with the data they need for CID resolution. It also allows for prefix and resource registration requests, as well as curation activities.

These Distributed Resolution services keep their working data in sync with the Central Registry by checking for updates regularly caching them locally in a Redis service for faster response times.

Graphical and programmatic access to these services is enabled by web frontends and a client library, cloud-libapi (https://github.com/identifiers-org/cloud-libapi).

## 3 Results

### 3.1 CID resolution

The Identifiers.org resolution system provides consistent access to Life Science data using CIDs. The resolving location of CIDs is determined using information that is stored in the Identifiers.org Registry. When a CID is presented to the Identifiers.org resolver, redirection can be accomplished in either a resource specified or location independent (resource unspecified) manner.

The Identifiers.org resolver selects the provider based on a resource recommendation index, which is calculated by a resource recommender microservice, which implements a scoring function that depends on factors like whether a resource is registered as the primary resource for that namespace, and a reliability score calculated by the link checking subsystem, which provides this scoring for every resource in the registry, based on periodical registry-wide resource link checking, and reactive resource link checks, upon resolution requests.

For example, NCBI is the primary resource for hosting Taxonomy, and it has a high reliability score, thus Taxonomy identifiers will be resolved to NCBI almost all the time unless the resource is down, or an alternative provider code is speficied.

### 3.2 Application interfaces for resolving CIDs

To assist users in adopting and using the Identifiers.org CID identifiers, we have developed a set of services to resolve CIDs and to register prefixes.

We have developed a responsive web front end that encompasses three different domains: (i) Resolver (https://identifiers.org/), which enables CID resolution in a visual manner, with a powerful auto-complete feature to guide the users through the construction of a CID; (ii) Registry (https://registry.identifiers.org/), featuring a browser for the full catalog of registered prefixes, and an improved prefix and resource registration request process with as-you-write validation features. (iii) Documentation pages (https://docs.identifiers.org/), a guide for users as well as a portal to the code repositories for developers interested in using the API.

The Identifiers.org metadata service enables users to access schema.org (https://schema.org/) and bioschemas (https://bioschemas.org/) metadata encoded in landing pages. Given a CID, this service will communicate with the resolution API to obtain the resolved resources, and then it will visit their URLs to extract JSON-LD formatted schema.org and bioschemas metadata.

An API has been developed to enable users to resolve CIDs and access Identifiers.org content programmatically. Services to view, search and request CIDs are also provided through the API (https://docs.identifiers.org/articles/api.html). We have developed a java client library for accessing Identifiers.org API Services. The java client library, org.identifiers.cloud is distributed via Maven Central (https://mvnrepository.com/artifact/org.identifiers.cloud). This can be used to programmatically specify and access the Identifiers.org CID resolver and registry.

## 4 Discussion

The new Identifiers.org cloud native platform supports over a million requests per month for 706 namespaces and 857 resources, with enough capacity for 10 times as much traffic before the need for scaling up the infrastructure, which can be done seamlessly. The uptime metric is above 99%, compared to the outages and technical problems the old one was prone to, and it leverages the cloud distributed presence and nature, as well as its internal networking optimization for lowering latency on services access.

The long-term commitment to maintaining an exhaustive and up to date list of active resolving locations is an expensive and often duplicated task; most publishers and data providers maintain such a registry to resolve to the most commonly referenced data resources to support internal linking, cross-referencing and resolution. Identifiers.org incorporates a centralized registry that actively curates such information, as well as providing prefixes for use within the Life Sciences community. Use of the Identifiers.org CID system facilitates the resolution and cross-referencing of concepts used within the domain, and reduces the costs of maintaining multiple such registries or lists.

Even though focused on Life Sciences, Identifiers.org CID resolution system and its infrastructure are generic enough to expand to other domains and systems, allowing the Identifiers.org system to resolve a wide variety of identifier schemes such as DOIs and ARKs by pointing to their meta resolvers.

We envisage the implementation of a cloud-based service infrastructure facilitates the adoption and use of Identifiers.org CIDs and related services by providing a robust, highly available and low-latency service.
